# Using citizen science for the energy transition: Research on the tenant electricity model in Germany

**DOI:** 10.12688/openreseurope.17722.1

**Published:** 2025-01-20

**Authors:** Johannes Baumann, Marcela Noreña, Pia Wieser

**Affiliations:** 1 Women Engage for a Common Future, Munich, Bavaria, 80331, Germany

**Keywords:** Renewable energy, citizen science, tenant electricity model, energy transition, neighbourhood energy sharing

## Abstract

**Background:**

The research within the Citizen Science (CS) project on tenant electricity focused on an inclusive research approach by involving actors such as citizen scientists (CSs), scientists, policymakers, and the private sector. The main objective was to jointly explore the barriers and drivers for and motivations to participate in the tenant electricity model in Germany, and to identify behavioural changes (based on the energy culture concept) of the CSs by being involved in local electricity production and consumption.

**Methods:**

The CS project adopted a mixed-method approach, combining qualitative data analysis from workshops with quantitative data from an energy consumption monitoring scheme and a panel survey on energy-related practices.

Results and conclusions: Identified barriers for the tenant electricity model encompassing both structural (e.g. model complexity) and inherent challenges (lack of information). Drivers for scaling up include the reduction of the complexity and bureaucratic hurdles of the model as well as regulations and financial incentives and targeted information to relevant actors.

The main motivation for participating in tenant electricity was sustainability and local production of electricity, while the electricity price played a minor role. Regarding changes in energy culture, the participation in tenant electricity led to a stronger exchange among neighbours about further sustainability options and to a higher interest in sustainability or society engagement.

Feedback on regular consumption data was perceived by almost all participants as useful for further measures to save electricity. Electricity data collected from installed meters showed, on average, a reduction in consumption for more than half of the households compared to the start month of the research period.

Further, a cluster analysis was conducted to identify different profiles and gain deeper understanding of the characteristics of CSs. In total five clusters were identified, with differences in energy consumption patterns, energy efficient appliances, knowledge about energy consumption, and changes in energy practices.

## Introduction

Citizen Science describes a phenomenon with a long tradition in scientific inquiry – the voluntary involvement of people not formally educated in the specific area of research, operating especially in data collection related to biodiversity and the environment. The stream of scientific research has been named “Citizen Science” in the 1990s and gained popularity and recognition by science and policy in the last decades ( Tweddle *et al.*, 2012). Nevertheless, it is important to highlight the scarcity of citizen science initiatives in the realm of renewable and decentralized energy generation. Regarding the context of the development of Citizen Science, Wuebben *et al.* (2020) highlight that one of the prominent representatives of citizen science research, Alan Irwin, developed the terminology and concept with respect to the 1987 UN Report “Our Common Future” ( Brundtland, 1987), where the idea of ‘sustainable development’ evolved ( Wuebben *et al.*, 2020). Given that “Citizen science and the sustainable development goals (SDGs) were born of the same moment” ( Wuebben *et al.*, 2020, p. 3), it becomes intriguing to consider which SDGs are addressed through Citizen Science projects. Wuebben *et al.* (2020) conducted a literature analysis showing that out of 127 Citizen Science projects in Germany, most tackled SDG 15 “Life on Land” and SDG 4 “Quality Education”, while none addressed SDG 7 “Affordable and Clean Energy”.

As renewable forms of energy bear the potential to make energy more accessible and inclusive to people and communities, which is also known under the term “democratization of energy”, it offers a paradigm change where citizen science can play a pivotal role for gaining new results.

Our research on clean energy applying a citizen science approach was conducted within the EU-funded *Step Change Project* ( https://stepchangeproject.eu/) *,* which builds on the assumption that Citizen Science can play an important future role by adding value to science and changing the way society views research. More precisely the research focused on the under-exploited potential of photovoltaic systems on multi-family buildings in Germany, mainly implemented through the so-called *tenant electricity* model. This model provides the opportunity for neighborhood-based electricity sharing with the sale of electricity generated directly within multifamily buildings with reduced fees and taxes ( Bundestag, 2017). As multi-family buildings accommodate around half of the German housing stock, the involvement of tenants in the production of clean electricity can be seen as key to boost the urban energy transition ( Moser *et al.*, 2021).

But realising that the tenant electricity model falls short of expectations and lacks broad citizen acceptance, interest arose in understanding the drivers and challenges of the tenant electricity concept. As part of the concepts ‘pushing’ for prosumer empowerment (as is the energy communities movement), the tenant electricity concept is dependent on the constant adjustments to its prosumer’s needs and criticism. To actively participate in these adjustment processes and serve as mediators and advocates for tenants, landlords, and political decision-makers, we decided to directly engage citizens as scientists.

In addition to the barriers and drivers for the tenant electricity model, the motivations of the citizen scientists (CSs) to participate in the tenant electricity model were examined. Furthermore, the initiative examined behavioural changes of the citizen scientists (based on the *energy cultur*e concept) due to their involvement in local electricity production and consumption. In detail, the five research questions are:

1. What are the *barriers and drivers* for involvement in tenant electricity models, and how can we overcome these barriers and accelerate drivers?
2. What *motivations* do citizen scientists have in participating in tenant electricity?
3. Does *participation in tenant electricity projects* have an impact on energy-related lifestyles and energy culture (e.g., self-efficacy: becoming more active in contributing to the energy transition, combating climate change)?
4. Does *regular data observation* about energy consumption have an impact on energy-related lifestyles and energy culture (e.g., energy-efficacy)?
5. Does *participation in this Citizen Science Project* has an impact on energy-related lifestyles and energy culture (e.g., energy-efficacy)?

## Methods

**Figure 1.**
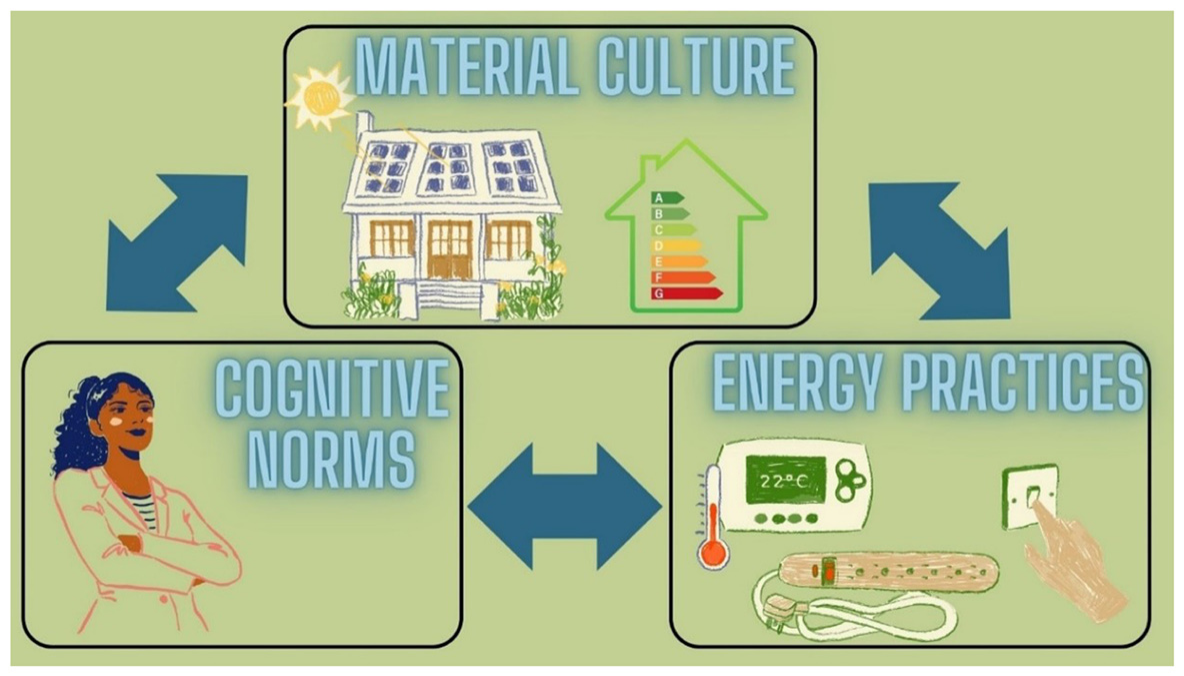
The three main pillars of Energy Cultures (image created by authors) adapted from: Stephenson *et al.*, 2010.

**Figure 2.**
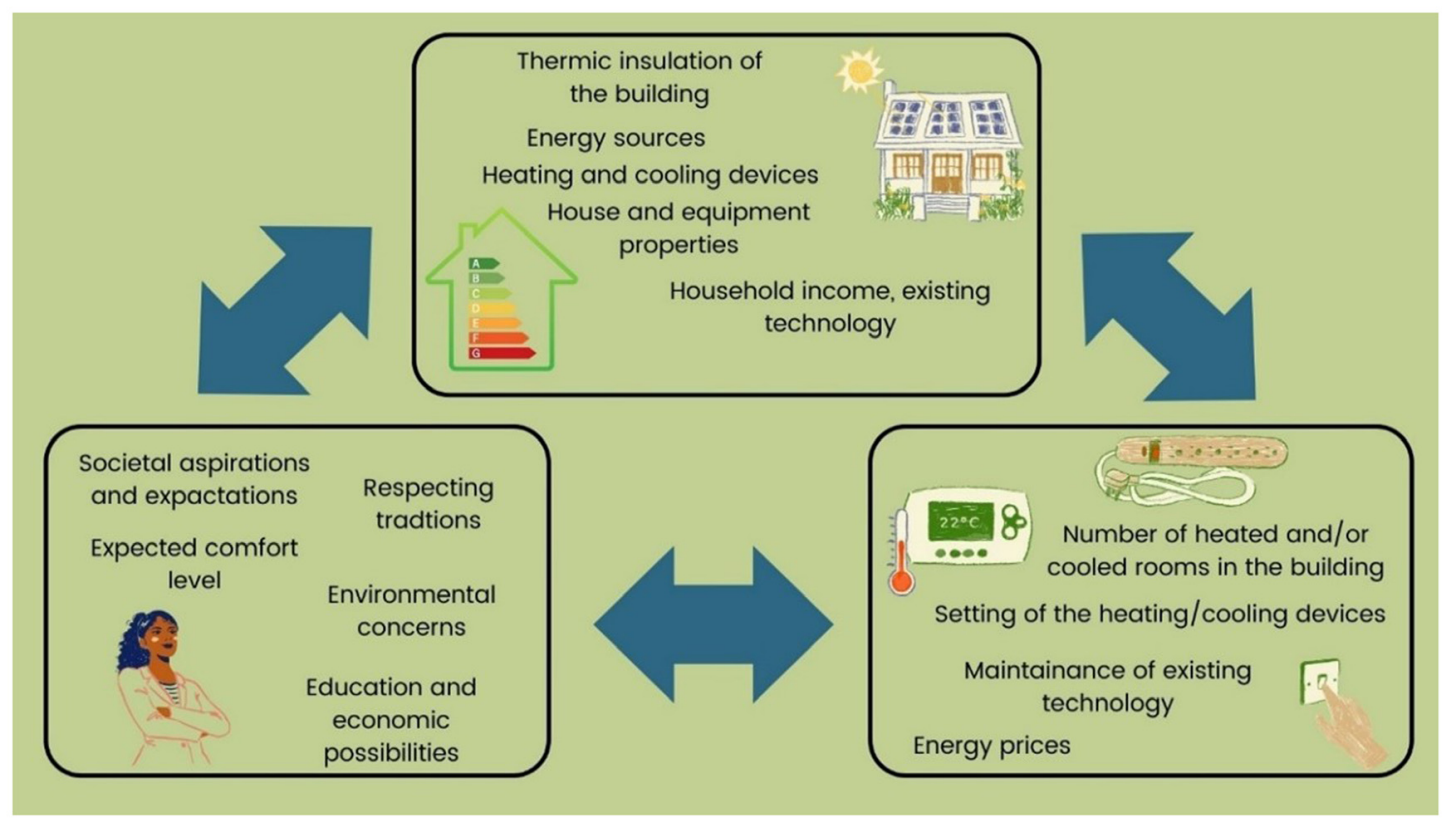
Examples of three main pillars of Energy Cultures (created by authors) adapted from: Stephenson *et al.*, 2010.

We have chosen the framework of *Energy Cultures* to analyze and understand the motivations behind changes in energy efficiency behaviors by attending to behavioral drivers ( Stephenson *et al.*, 2010). The Energy Cultures framework was developed as an integrating model highlighting explanations of behavioral change or resistance to change and consequent solutions nudging the transformations of consumer habits. The three main pillars that characterize the Energy Cultures framework – norms, material culture, and energy practices– interact and influence each other ( Figure 1, Figure 2). For example, one’s socialization (norms) affects technological preferences (material culture) and activities (energy practices). External circumstances also play an important role (e.g. price shocks) in influencing energy behaviour alongside cognitive norms, material culture, and energy practices ( Stephenson *et al.*, 2015). The framework's core hypothesis is “that stabilization of behaviour occurs, where norms, practices and technologies are aligned – that is, where the dynamics between the three components are self-reinforcing. Potential for behaviour change arises when one of these components becomes misaligned or shifts […].” ( Stephenson *et al.*, 2010, p. 6125).

**Figure 3.**
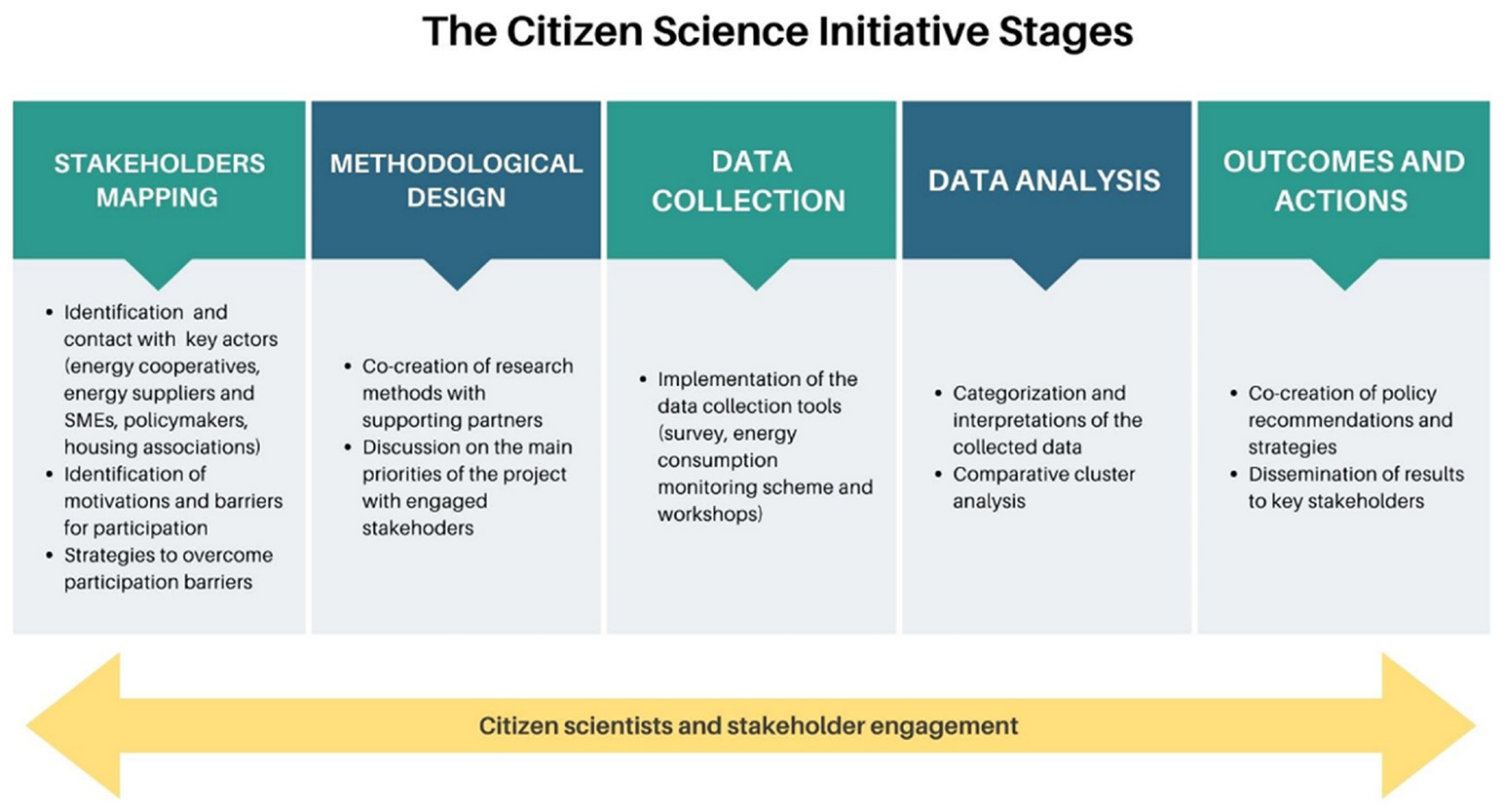
Stages of the citizen science initiative on tenant electricity (Authors’ own elaboration).

The research design of our Citizen Science project consisted of five main stages starting with stakeholder mapping and recruitment, elaborating the methodological design, conducting data collection and analyses and dissemination of recommendations, as shown in Figure 3.

After recruiting 37 citizen scientist households, the core research team collectively designed the data collection tools and protocols. The citizen science initiative adopted a mixed-method approach, combining qualitative data analysis from three workshops and quantitative data from an energy consumption monitoring scheme and a panel survey on energy-related practices.

Before starting the data collection phase, citizen scientists received either access to software for monitoring energy consumption or intelligent meters were installed in their homes. Citizens were trained in the use of the software and intelligent meters through an online workshop. In addition, two virtual meetings (Kick-off meetings) were held to inform citizens about the project objectives, methodologies, and opportunities for contributions beyond home data collection.

The active data collection phase was launched with the distribution of the first of three surveys, forming the baseline for analyzing changes in practices, attitudes, and motivations at home. Upon concluding the first round of surveys, the first online workshop aimed to deepen the understanding of perceptions of and experiences with the tenant electricity model.

Concurrently, the collection and reporting of household energy consumption started. Participants were asked to report only their monthly total consumption via an online platform. However, the software installed in the framework of the citizen science initiative allowed them to track other consumption statistics such as hourly and daily energy consumption.

**Figure 4.**
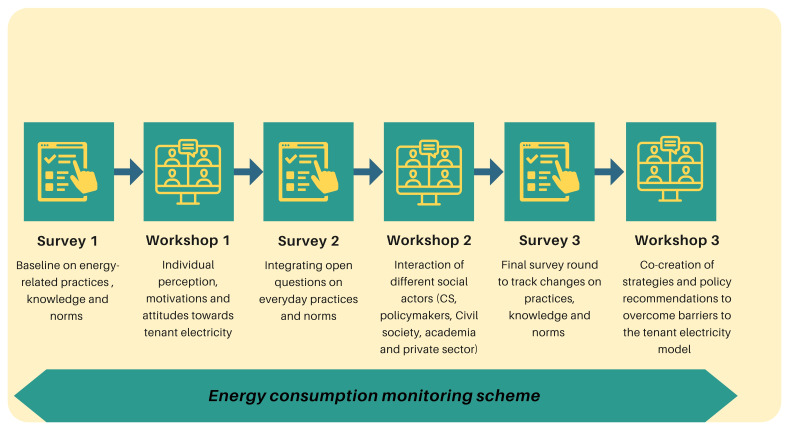
Data collection methods (authors’ own elaboration).

This structure (conducting a workshop after each round of surveys) was carried out three times in total. The workshops offered a platform to share the preliminary results of the survey with citizens, to delve deeper into issues of interest (thus increasing energy literacy), and to generate discussion on the various challenges and barriers encountered by the tenant electricity model and community energy more broadly within the local and national context. Figure 4 provides additional information on the data collection tools.

All research methods were applied between September 2022 and May 2023. After each workshop, the core research team analyzed the collected data, compiling thematic summaries with the most salient information. Together with the preliminary results of the survey, these results were shared and discussed with citizen scientists, experts, politicians and the private sector. This activity also allowed to gather information about their perceptions and expectations regarding the citizen science initiative and its impact.

Upon completion of the third survey round, the analysis stage continued with a comparative analysis and hierarchical clustering, aiming at identifying how the energy culture of the citizen group changed during the citizen science initiative and creating consumer profiles.

## Results and discussion

### Barriers of the tenant electricity model

Most of the current studies on tenant electricity often focus on barriers to the tenant electricity model resulting from regulatory frameworks that end in technical and economic barriers (e.g. Moser *et al.*, 2021). However, a study on the acceptance of tenant electricity from the perspective of tenants ( Schäfer, 2019) found that aspects related to lower costs, the provision of renewable energy, and sustainability concerns are key determinants of a tenant's decision to participate in tenant electricity. Our research builds on this study and provides new insights into the barriers identified. We identified a number of barriers which encompassed both structural and inherent challenges. Structural barriers were:

- Lack of (former) political willingness to promote the model
- Complexity of the model
- Low economic incentives to implement the model on a broader scale

While the inherent barriers were:

- Lack of *information* about the model on all levels and
- Lack of *initiators* who are able to drive the implementation of the model at the local level.

### Strategies for overcoming barriers

Overcoming barriers implies measures and strategies from different actors such as political actors, energy supply actors and building-related actors, such as residents and building-owners.

One of the essential steps for scaling up the model is its simplification, and political actors are responsible for reducing the complexity of the model. There are some noticeable developments in this regard as the current Federal Government in Germany is planning to include strategies for bureaucracy reductions and further development of the existing tenant electricity model as a component of its photovoltaic strategy ( Bundesministerium für Wirtschaft und Klimaschutz, 2023, pp. 18 – 22 ). Moreover, the new strategy considers the introduction of community supply within a building (proportionate allocation of generated pv electricity to residents resulting in a reduction of grid electricity), which would be an additional entry point for the implementation of energy sharing options in buildings.

Policymakers can also use regulations to encourage the expansion of tenant electricity, for example by requiring the installation of photovoltaic panels on new buildings and requiring grid and metering operators to apply a standardised metering concept. Economic incentives could not only be part of the model itself, but local authorities can support special programmes and use their scope for action. This requires a proactive role for local authorities in informing and reaching out to residents, e.g. by checking the photovoltaic cadastre and approaching property owners.

**Energy supply actors,** such as utilities and tenant electricity providers, can contribute to faster uptake through targeted information and marketing to relevant audiences, such as property managers, housing cooperatives and energy cooperatives. Prospective customers in multi-family dwellings can be reached by advertising the tenant electricity model in their bills. In addition, energy companies have a financial control instrument if they offer a two-tariff system and provide cheaper electricity when it is produced by the PV system.

Concerning the lack of initiators at the local level, (also) **residents** can contribute to a faster uptake of the tenant electricity model. The exchange with interested neighbours and the promotion of the project at owners' meetings are key for a bottom-up approach. The necessary support from apartment owners can be achieved by demonstrating the potential increase in property value by using PV systems.

### Motivation and sustainability awareness of citizen scientists

Another objective of our research was to identify motivation of citizen scientists participating in tenant electricity. Data from the workshops and surveys broadly align with previous observations (e.g., Schäfer, 2019), linking participation in tenant electricity to sustainability awareness and to a lesser extent to the potential lower electricity prices.

**Figure 5.**
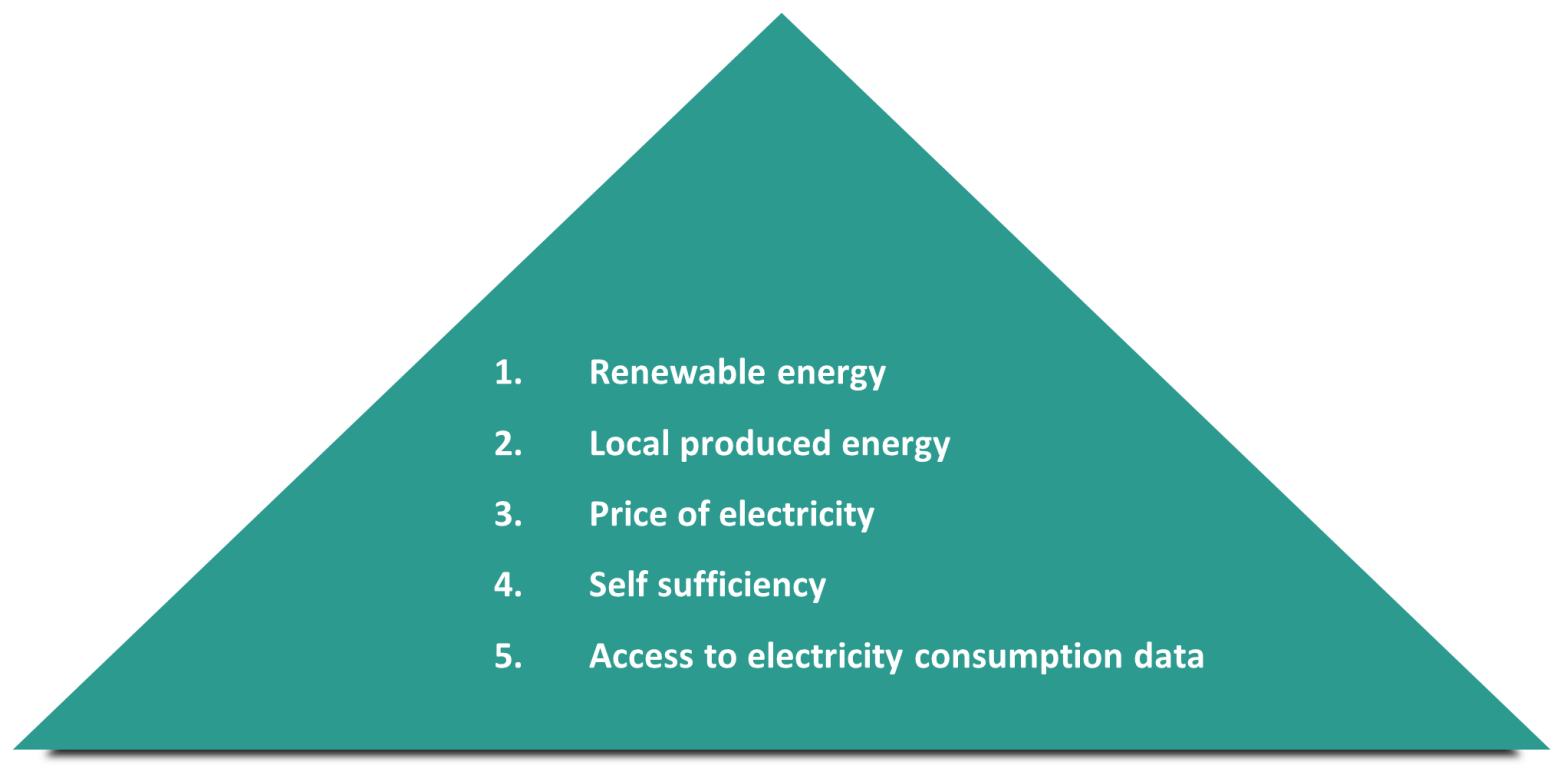
Ranking of important aspects for the Citizen Scientists (own source).

Surprisingly, despite the general increase in electricity prices due to Russia's invasion of Ukraine, participants in the citizen science initiative still appear to have low price sensitivity. These findings provide support for the notion of *changing social norms* as a key determinant of energy behaviour and a more sustainable energy culture (see Figure 5).

On the other hand, for almost a fifth of the participants, the main motivation for participating in tenant electricity was the availability and promotion of the model in the building. However, it should be noted that 70% of the citizen scientists live in housing cooperatives where the implementation of rental electricity was decided by a simple majority at the general assembly meeting. However, the conclusion of a tenant electricity contract is in any case voluntary. This finding indicates that, taking into account the specific composition of our group, the bottleneck in the implementation of tenant electricity is not due to a lack of demand.

### Impact on energy culture

A possible change in energy culture was examined through the participation in tenant electricity, the feedback on regular electricity consumption data, and by the participation in this research project. The participation in tenant electricity has led to an overall stronger exchange among neighbours about further sustainability options in the building and to higher interest in sustainability actions with friends and family. In general, the citizens scientists have expressed interest in participating in social and environmental initiatives, including energy cooperatives and associations with a sustainability focus. Similarly, participation in tenant electricity also spills over in interest in conducting sustainability activities at the workplace. This indicates a general positive impact on the energy culture.

**Figure 6.**
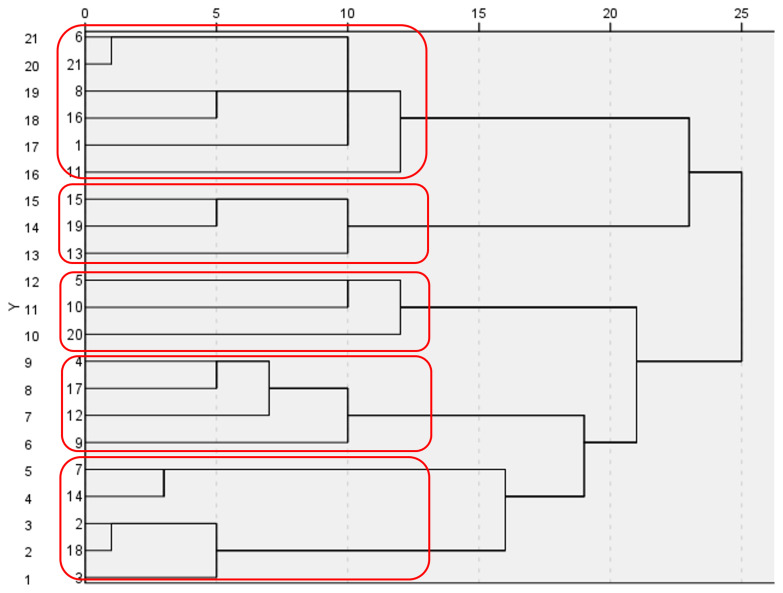
Dendrogram of clustering process with 5 clusters. The clusters represent different sub-energy cultures by showing differences in energy consumption patterns, energy efficient appliances, knowledge about energy consumption, and changes in energy practices (own source).

A detailed cluster analysis was conducted to find different profiles and gain a deeper understanding of the characteristics of the citizen scientists. In total 5 clusters were identified, with differences in energy consumption patterns, energy efficient appliances, knowledge about energy consumption, and changes in energy practices due to participation in tenant electricity and the research project ( Figure 6).

Especially one cluster stands out when referring to the changes caused by the *participation in tenant electricity*. For them, participation led to further interest or action in nearly all the above-mentioned areas. This is of special interest as people of this cluster had the highest relative energy consumption of all five clusters, an overall low material culture and low knowledge about classifying the electricity consumption in regard to comparable households. As the cluster covers nearly 20% of the participating citizen scientists, it is diverse in income and household situation it can be seen as the group where tenant electricity did the most to reflect about and change their energy culture.

### Feedback on regular consumption data and energy culture impacts

Analysing the feedback on regular consumption data regarding impacts on energy culture, most participants found it useful for further action to save electricity. According to the survey participants’ responses, the regular review of energy use through the metering software has led to a reduction in energy consumption through behavioural change for around a quarter of the citizen scientists. When it comes to purchasing new energy efficient equipment, only a small share of the citizen scientists expressed that feedback on energy consumption motivated them to change their equipment.

Backing up the survey results with electricity consumption data during the research period, it was visible that more than half of the households used less energy on average during the research period compared to the starting month of the research period. Only six households used more electricity during the data collection phase, but it must be acknowledged that five of these households still used less energy than an average comparable German household.

Additional information about the type of electricity available - solar from the roof or from the grid - and the ability to compare with other households (“gamification”) were seen as drivers to encourage more conscious use of electricity. This should be taken into account by tenant electricity suppliers when providing metering software to their customers.

### The citizen science project and its impact on energy culture

The final research question related to the impact of participation in a citizen science project on energy culture. In this regard, we initiated regular electricity tracking for about half of the citizen scientists, which can be related to higher awareness on the own consumption and foster behavioral changes in the long run. Participants also became more aware of energy efficient appliances and around a quarter reported changes in certain energy-intensive practices (e.g., mobility or flights). Similarly, the project also initiated the use of ecological footprint calculators for about one third of the citizen scientists.

## Conclusion

Within our research, we were able to shed light on each research question. While the barriers are mainly of structural nature it was surprising that knowledge and information about the tenant electricity model is lacking on multiple levels. Targeted information campaigns could solve the latter while political efforts must be made to overcome the structural barriers. Surprisingly, the main motivation for participating in tenant electricity was sustainability and local production of electricity while the price of electricity played a minor role. The influence on energy related lifestyles and energy culture was multidimensional and resulted from the participation in tenant electricity projects (e.g. exchange with neighbours about sustainability options in the building), the feedback on electricity consumption data (leading to a reduction in electricity consumption), and the participation in our citizen science project (e.g. getting access to the intelligent metering system, getting aware of energy efficient appliances).

The cluster analysis also provided interesting research opportunities, as each of the five elaborated clusters had their own sub-energy culture determined by energy consumption patterns, equipment with energy efficient devices, knowledge on energy consumption and energy practice changes. A better understanding of each cluster could be conducive for a more targeted interventions and nudges that encourage citizens to participate in energy sharing models and to change energy-related behaviours.

There is also a growing need for research into the social justice implications of the tenant electricity model and, more generally, of community energy and energy sharing. Our findings show that citizen science has the potential to help analyse whether these models provide equal access and benefits across different demographic groups and socio-economic backgrounds, and how gaps can be addressed.

Finally, it should be noted that the conducted research can be seen as a pioneer project on the implementation of a Citizen Science project in the area of sustainable goals SDG7 (clean energy) through the analytical lens of the energy cultures framework, and that it will lay the groundwork for further citizen science and institutionalised science to expand and complement.

We find ourselves in a dynamic phase with fundamental legal, bureaucratic, and financial changes and development of the tenant electricity model through the current government, which brings further research opportunities regarding the model. Since the motivation to participate in tenant electricity is highly influenced by sustainability concerns, it is relevant to conduct further research on the ways to use changing social norms towards sustainable behaviour to increase the acceptance of tenant electricity and energy sharing models.

## Ethics and consent

In accordance with the ethical and data protection principles agreed upon for the Step Change project, we provide the following statement on participant consent:

**Consent type and justification:** The study utilizes data that were collected as part of an earlier research phase within the Step Change project. During this initial phase, participants provided written consent for the collection, use, and analysis of their data, with the understanding that any data shared or published would be anonymized. As such, explicit consent for this particular publication was not separately obtained, as it falls within the scope of the original consent provided by participants.

**Ethical approval:** Ethics approval was received from the WECF Ethics Committee, led by Dr. Anke Stock, responsible for overseeing the Step Change project on 1 ^st^ April 2022. The committee confirmed on 15 ^th^ July 2024 that the publication adheres to the ethical standards and data protection principles established for the project. After careful consideration, the management team, in consultation with the ethics officer, determined that additional consent from participants was not required for this publication. This decision was based on the fact that the data used in this study is anonymized and participants had already agreed to such usage in their original consent.

**Ethics committee review:** The decision to waive the need for additional consent was reviewed and approved by the ethics officer overseeing the project. The officer supported this approach, recognizing that the original consent covered the anonymized use of data for future analyses and dissemination within academic and analytical contexts.

Should you have any further questions regarding the ethical considerations or consent procedures for this study, please contact the project’s ethics officer, Dr. Anke Stock, at anke.stock@wecf.org

## Data Availability

The energy consumption data collected from participating households is considered highly sensitive. To ensure the privacy and confidentiality of participants, access to the raw data has been restricted exclusively to the researchers from WECF involved in this study. As a result, only aggregated data will be available for external use. The study, including its data management, was reviewed by the WECF ethics officer. The WECF Ethics officer emphasized the importance of protecting participants' privacy and endorsed the decision to limit data access to aggregated datasets only. They also supported the case-by-case evaluation of requests for data access, ensuring that any sharing of data aligns with ethical standards. Researchers or reviewers interested in accessing further aggregated data may contact the project manager, Johannes Baumann, at johannes.baumann@wecf.org. Applications for data access should include a detailed explanation of the legitimate interest in the data and how it will be used. The project manager and ethical officer will assess each request individually, considering the relevance and purpose of the research. If approved, access will be granted under specific conditions, which may include agreements on data usage, confidentiality, and publication rights.
