# Supplementary material for: Using citizen science for the energy transition: Research on the tenant electricity model in Germany
Source: Open Res Eur. 2025 Jan 20;5:14. [Version 1] doi: 10.12688/openreseurope.17722.1 (PMC11883211; doi:10.12688/openreseurope.17722.1)
Supplement: Peer review history

# Reviewer report: Reviewer response for version 1

**DOI:** 10.21956/openreseurope.19156.r50382

**Published:** 2025-03-05

**Authors:** Bożena Ryszawska

**Affiliations:** 1 Wroclaw University of Economics, Wroclaw, Poland

It seems interesting to me to include citizens scientists in energy transition research. The choice of a research approach using citizen science highlights the roles of citizens and not just consumers in the energy transition. Current European Union policy focuses on the market and on two actors: the producer and the consumer. People are reduced to the role of consumer, they are assigned consumer standards of behaviour and motivation, e.g. energy is a commodity, cost reduction, low price, and access to the grid. 

 The positive results of research using citizen science are related to:

- empowering citizens in the study,
- giving them an important role as co-researchers,
- noticing their expertise in the subject,
- through workshops making information and research results available to them on an ongoing basis. 

The conclusions of the research are very interesting: structural (e.g. model complexity) and inherent challenges (lack of information). These conclusions are confirmed by many other studies. Citizens as transformation actors are placed on the market in a complex model and are confronted with the necessity of professional knowledge in management, finance and law as well as engineering. They are forced to compete with other energy producers and consumers (large energy corporations, manufacturing companies, etc.). The market structure, regulations, financial tools, access to information, support from local government are not adapted to the needs of citizens.  Therefore, the authors advocate the reduction of the complexity and bureaucratic hurdles of the model as well as regulations and financial incentives and targeted information to relevant actors.

 The value of the research is to draw attention to energy culture. Because in reality, energy for citizens is not just a commodity, it is also a natural resource, a social necessity and even a common good. Therefore, it emerged in the motivations of the respondents that price reduction is not the most important motivation. What the research participants pointed out was: participation in tenant electricity led to a stronger exchange among neighbours about further sustainability options and to a higher interest in sustainability or society engagement. 

 An important element of the research was the observation that: multi-family buildings accommodate around half of the German housing stock, the involvement of tenants in the production of clean electricity can be seen as key to boost the urban energy transition’. To date, the energy transition has been designed more with single-family homes in mind, often meaning support for wealthier individuals rather than energy justice for all.

 I suggest to the authors that the study should be complemented by a context of citizens and indicate that citizens are not systematically supported in the energy transformation process.

If applicable, is the statistical analysis and its interpretation appropriate?

Not applicable

Is the study design appropriate and is the work technically sound?

Yes

Is the work clearly and accurately presented and does it engage with the current literature?

Partly

Are the conclusions drawn adequately supported by the results?

Yes

Are sufficient details of methods and analysis provided to allow replication by others?

Yes

Are all the source data and materials underlying the results available?

Yes

Reviewer Expertise:

Sustainability transition, citizens energy transition, green economy, sustainable finance

I confirm that I have read this submission and believe that I have an appropriate level of expertise to confirm that it is of an acceptable scientific standard.

# Reviewer report: Reviewer response for version 1

**DOI:** 10.21956/openreseurope.19156.r50390

**Published:** 2025-03-03

**Authors:** Silvia Tomasi

**Affiliations:** 1 Institute for Renewable Energy, Eurac Research, Bolzano, Italy

The research idea is interesting, but the paper requires major revisions to meet scientific quality standards. To begin with, the background literature review is insufficient: several key concepts are introduced without proper references, and their connections are presented in a confusing manner. This suggests a lack of a comprehensive literature review, which needs to be addressed to strengthen the paper’s theoretical foundation. Scientific references and explanations of key concept of the research are misplaced in the methods and results sections.

 Overall, the study sets too many objectives (and possibly employs too many data collection methods), resulting in superficiality and confusion in the findings. The research design could have been structured into three distinct papers, each with a clear focus: one examining changes in consumption behaviors resulting from participation in tenant electricity programs, another identifying barriers and drivers of participation through workshops with diverse stakeholders, and a third exploring the impact of citizen science projects on energy consumption behaviors. This approach would allow for a more in-depth and coherent analysis of each topic.

 Finally, the most worrying aspect is that while the data collection methods are mentioned (though not comprehensively—see my comment below in the dedicated section), the authors do not explain the methods used to analyze the collected data. This omission not only hinders the reproducibility of the study but also raises concerns about the reliability and validity of the results. 

 Hereafter are some specific revisions. I stopped before completing the *Results and Discussion* section because the study contains too many flaws.

 Abstract: 

 The abstract has to be deeply reviewed. 

 The first paragraph of the abstract is oddly structured. Please, first provide the context for the study, and the main research gap in addresses. Then clarify the aim of the study. In the first paragraph of the abstract there is also a reference to the concept of energy culture, please integrate a brief definition of it. 

 Results in the abstract are too synthetic and thus not fully comprehensible (e.g. what is meant with tenant electricity model? The energy consumption scheme citizens are involved in?).

 Introduction:

 Include a reference (there is plenty in the literature) for the paragraph stating “As renewable forms of energy bear the potential to make energy more accessible and inclusive to people and communities…”. You can further elaborate on this step in your narrative by strengthening the connection between the concept of citizen science and that of energy democracy.

 Be consistent with terminology: either you chose to use the term “renewable energy” or “clean energy”. 

 The following statement needs to be referenced: “But realizing that the tenant electricity model falls short of expectations and lacks broad citizen acceptance”. This is a gap in the literature that your research aims to address, and it needs a strong foundation in the existing literature.

 The following sentence is not clear: “As part of the concepts ‘pushing’ for prosumer empowerment (as is the energy communities movement), the tenant electricity concept is dependent on the constant adjustments to its prosumer’s needs and criticism.” You introduce two different concepts (prosumer empowerment and energy communities) without any scientific reference and without explaining how they are connected to the tenant electricity concept (you state this last is part of the former two…). Moreover, the syntax of your statement needs to be revised to enhance clarity and readability.

 The citizen scientists you engaged are the same citizens involved in the tenant electricity model? All of them or just some of them? 

 The introduction of the concept of energy culture needs both scientific references and a short explanation of what it entails. Such description and reference is reported later in the Methods section, please move it to the Introduction section. 

 This section in general lacks information about existing literature (and there is plenty) concerning barriers and drivers for citizens involvement in prosumers schemes (what you call tenant electricity model, which I would be interested to understand if it is called like that just in Germany and what are the difference with other citizens engagement approaches in prosumers schemes), motivation leading to citizens involvement in tenant electricity models (which form my point of view overlaps with the drivers mentioned before, I urge you to explain which is the difference), effects of participation in the model and energy data observation on energy consumption behaviors. Finally I do not fully understand the difference between RQ 3 and 5: not all citizens participating in the tenant electricity project (seek for consistence between the different terminology you use: tenant electricity project and tenant electricity model) are also participating as citizen scientists in your project? 

 Methods:

 The first paragraph concerning the energy culture framework is misplaced, should be located in the introduction where you discuss the main concepts and reference literature. 

 It is not entirely clear why the authors refer to citizen science and citizen scientists in their study, given that their research design primarily involves collecting household energy consumption data through software or smart meters, supplemented by surveys and workshops examining changes in consumption behavior. In this context, participants appear to be subjects of the research—whose behavioral changes are being investigated after receiving information—rather than actively contributing to shaping the research questions or methodology, as is typically expected in citizen science initiatives. Clarifying this distinction would strengthen the conceptual alignment of the study.

 There is a mismatch between the text and the content of figure 4: in the text you just mention citizens as participants to the workshops, while from the figure it emerges that other stakeholders participated in some of the workshops. 

 There is no reference on how the data collected has been analyzed. Both the analysis of qualitative data should be thoroughly explained (e.g. thematic analysis) and also quantitative models (e.g. regression). 

 Results

 Here too you included references to the literature which should have been placed in the introduction section (or even better in a dedicated literature review section). The results and discussion section should first report the results of your analysis (in this case of both quantitative and qualitative data) and then discuss them in light if the existing literature. 

 You report your results on barriers, which if I correctly understood emerged from the discussion in one of the workshops, but you do not provide sufficient information to fully understand them: who identified these barriers? Citizens? Other stakeholders from the public and private sector? 

 Also results concerning the engagement in citizen science are not supported by thorough explanation.

If applicable, is the statistical analysis and its interpretation appropriate?

No

Is the study design appropriate and is the work technically sound?

No

Is the work clearly and accurately presented and does it engage with the current literature?

No

Are the conclusions drawn adequately supported by the results?

No

Are sufficient details of methods and analysis provided to allow replication by others?

No

Are all the source data and materials underlying the results available?

No

Reviewer Expertise:

Environmental economics, applied econometrics, energy transition, energy and environmental justice, socio-ecological transformations

I confirm that I have read this submission and believe that I have an appropriate level of expertise to state that I do not consider it to be of an acceptable scientific standard, for reasons outlined above.

# Reviewer report: Reviewer response for version 1

**DOI:** 10.21956/openreseurope.19156.r50386

**Published:** 2025-02-27

**Authors:** Jussi Valta

**Affiliations:** 1 Tampere University, Tampere, Finland

Thank you for allowing me to comment on the manuscript. It is an interesting work with a mixed method approach, which allows to triangulate and find credible results. I think the results are interesting, and also partly surprising if I understood them right.

 This part: *"This is of special interest as people of this cluster had the highest relative energy consumption of all five clusters, an overall low material culture  and low knowledge about classifying the electricity consumption in regard to comparable households."*

 Would be interesting to hear from you why this might be the case.

 Overall, the manuscript needs a clear description of the case and the sample that participated in the study. This could include data on where the building is, what energy it uses, apartment ownership profiles, income levels/ is it a prosperous area,...)  

 The basic notions of the data should be described more clearly: What was asked and possibly, how many responded, what were the means, standard deviations (when applicable), what were interview questions.

 In many cases, the results are not backed by numbers or quotations, which leaves the reader wondering on what kind of data are they based on and how the data was analysed.

 The cluster analysis seems interesting. You could add the names and descriptions for each cluster and descriptions on the "sub-energy cultures" you mention.

 The methods and data was not presented in detail it is hard to assess its reliability and validity.

 The recommendations for different actors could be more towards the end. I felt that these were more based on authors' ideas and not the surveys. This division of discussion and results should be cleared.

 Small notion: in the sentence *"We find ourselves in a dynamic phase with fundamental legal, bureaucratic, and financial changes..."* the word fundamental seems a strong word. Maybe back it up or change it?

 For many readers, the German Tenant electricity model might be unfamiliar. Description of its principles and functions would clarify a lot. Also, there is a notion *"But realising that the tenant electricity model falls short of expectations and lacks broad citizen acceptance, interest arose in understanding the drivers and challenges of the tenant electricity concept. " *Is there some reference to this statement?

 Overall, it is a nice research, but the research process should be more transparent. You could also discuss how the different research approaches complement each other.

If applicable, is the statistical analysis and its interpretation appropriate?

Partly

Is the study design appropriate and is the work technically sound?

Partly

Is the work clearly and accurately presented and does it engage with the current literature?

Partly

Are the conclusions drawn adequately supported by the results?

Yes

Are sufficient details of methods and analysis provided to allow replication by others?

No

Are all the source data and materials underlying the results available?

Partly

Reviewer Expertise:

NA

I confirm that I have read this submission and believe that I have an appropriate level of expertise to confirm that it is of an acceptable scientific standard, however I have significant reservations, as outlined above.

# Reviewer report: Reviewer response for version 1

**DOI:** 10.21956/openreseurope.19156.r50387

**Published:** 2025-02-19

**Authors:** Ogechi Vivian Nwadiaru

**Affiliations:** 1 University of Massachusetts, Amherst, USA

**Engagement with Current Literature**

 The manuscript addresses a pertinent issue related to tenant electricity and citizen science, which is highly relevant to the ongoing discourse on accelerating the energy transition. However, the support from existing literature is insufficient. The introduction does not fully justify the role of citizen science in the tenant electricity model or explain how it could contribute to advancing energy democracy. Additionally, the authors should provide more context on prior research in this area and clarify how this study builds on or extends previous work.

**Study Design**

 The study design appears appropriate. However, important details are missing, such as how citizen scientists were recruited, how engagement with the study community was facilitated, and the geographical scope of the research. Furthermore, the demographics of the participants are not clearly outlined, which limits the ability to assess the representativeness and applicability of the findings.

**Replicability**

 The authors provide a useful figure illustrating the stages of a citizen science initiative. However, there is insufficient detail on the data collection tools, analysis methods, and thematic coding process. Several key questions remain unanswered:

- Was a codebook developed? If so, how?
- Have the citizen scientists had prior exposure to sustainability topics?
- How was the survey instrument developed?
- Can the survey instrument be included as supplementary material to help reviewers better understand the questions covered?

Additionally, references to existing literature that informed the survey design and workshop methodology should be included.

**Results**

 The results section lacks contextualization through direct quotes from survey responses or thematic analysis. Providing evidence to support the claims in the results would enhance clarity and credibility. For instance:

- How were structural barriers identified?
- How many respondents or participants perceived model complexity as a structural barrier, and how did they define it?
- Discussing barriers and drivers would benefit from direct quotes, as is common in qualitative research.

Moreover, Figure 5 requires further explanation. What do the rankings illustrate? A brief description should be added to clarify their significance.

**Overall Assessment**

 The manuscript would be significantly strengthened if the authors address the issues outlined above. Additionally, the research questions should be more concise, and the results section should explicitly address each question.

 Furthermore, several key concepts—such as energy democracy, energy literacy, and energy cultures—are introduced without adequate explanation. The authors should clarify how these concepts relate to one another and to the tenant electricity model, using references from existing literature to support their discussion.

If applicable, is the statistical analysis and its interpretation appropriate?

Not applicable

Is the study design appropriate and is the work technically sound?

Partly

Is the work clearly and accurately presented and does it engage with the current literature?

Partly

Are the conclusions drawn adequately supported by the results?

No

Are sufficient details of methods and analysis provided to allow replication by others?

No

Are all the source data and materials underlying the results available?

No

Reviewer Expertise:

Community based participatory research, optimization, energy systems modelling and energy justice

I confirm that I have read this submission and believe that I have an appropriate level of expertise to confirm that it is of an acceptable scientific standard, however I have significant reservations, as outlined above.
